# Supplementary material for: Transferrin-Decorated PLGA Nanoparticles Loaded with an Organoselenium Compound as an Innovative Approach to Sensitize MDR Tumor Cells: An In Vitro Study Using 2D and 3D Cell Models
Source: Nanomaterials (Basel). 2023 Aug 11;13(16):2306. doi: 10.3390/nano13162306 (PMC10458402; doi:10.3390/nano13162306)
Supplement: Supplementary file 1 [file nanomaterials-13-02306-s001.zip › nanomaterials-2498986-supplementary.pdf]

nanomaterials

MDPI

Supplementary Materials

Transferrin-decorated PLGA nanoparticles loaded with an organoselenium compound as an innovative approach to sensitize MDR tumor cells: an *in vitro* study using 2D and 3D cell models

Letícia Bueno Macedo [1], Daniele Rubert Nogueira-Librelotto[1,2], Daniela Mathes[1], Taís BaldisseraPieta[2], Micheli Mainardi Pillat[1,3], Raquel Mello da Rosa[4], Oscar Endrigo Dorneles Rodrigues[4], Maria Pilar Vinardell[5,*], Clarice Madalena Bueno Rolim[1,2,*]

1 Programa de Pós-Graduação em Ciências Farmacêuticas, Universidade Federal de Santa Maria, Av. Roraima 1000, 97105-900 Santa Maria, RS, Brazil
2 Departamento de Farmácia Industrial, Universidade Federal de Santa Maria, Av. Roraima 1000, 97105-900 Santa Maria, RS, Brazil
3 Departamento de Microbiologia e Parasitologia, Universidade Federal de Santa Maria, Av. Roraima 1000, 97105-900 Santa Maria, RS, Brazil
4 Departamentode Química, Universidade Federal de Santa Maria, Av. Roraima 1000, 97105-900 Santa Maria, RS, Brazil
5 Departament de Bioquimica i Fisiologia, Facultat de Farmacia i Ciències de l'Alimentaciò, Universitat de Barcelona, Av. Joan XXIII 27-31, 08028 Barcelona, Spain
* Correspondence: mpvinardellmh@ub.edu (M.P.V.); clarice.rolim@ufsm.br (C.M.B.R.)

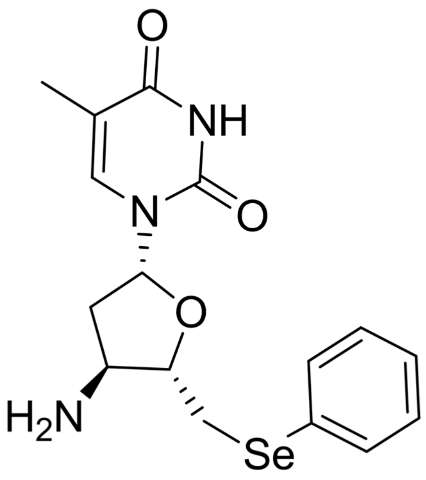

Figure S1. Chemical structure of 5′-Se-(phenyl)-3-(amino)-thymidine.

Table S1. Characterization of Tf-ACAT-Se-PLGA-NPs.

	Tf-ACAT-Se-PLGA-NPs			Mean	SD
Particlesize (nm)	121.3	148.1	142.1	137	5.48
PDI	0.128	0.103	0.151	0.127	0.02
ZP (mV)	-4.17	-3.64	-5.28	-4.36	0.82
pH	7.52	7.6	7.03	7.38	0.29

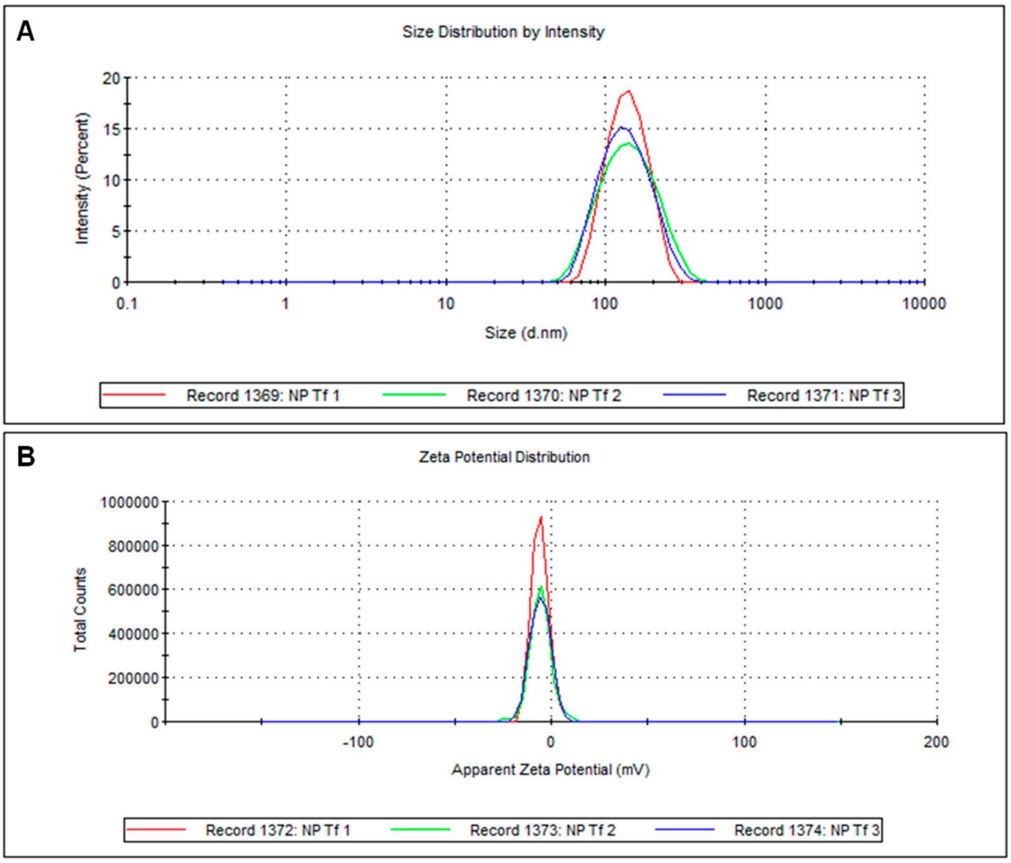

Figure S2. Characterization of Tf-ACAT-Se-PLGA-NPs determined by dynamic light scattering. Size distribution (A) and zeta potential (B).

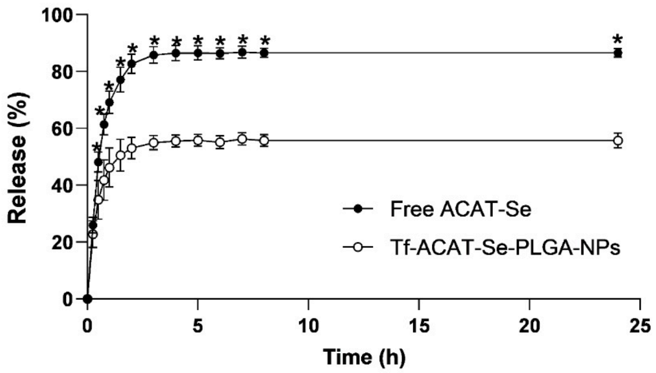

Figure S3. *In vitro* cumulative ACAT-Se release. Statistical analyses were performed using ANOVA followed by Tukey's multiple comparison test. *Significant difference from Tf-ACAT-Se-PLGA-NPs ($p < 0.05$).

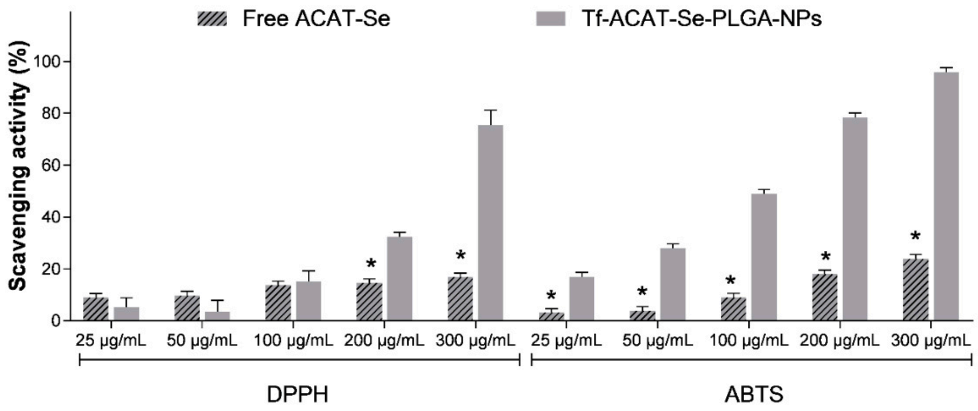

Figure S4. Scavenging activity of free ACAT-Se and Tf-ACAT-Se-PLGA-NPs by DPPH and ABTS assays. Results are expressed as mean ± SD of three independent experiments. Statistical analyses were performed using ANOVA followed by Student–Newman–Keuls multiple comparison test. *Significant difference from Tf-ACAT-Se-PLGA-NPs ($p < 0.05$).

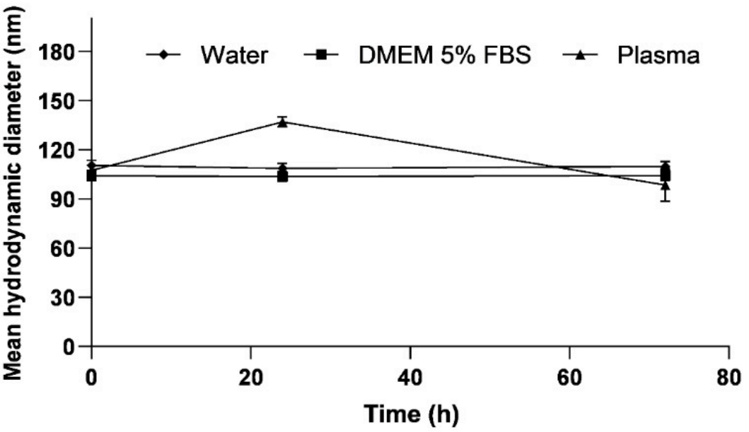

Figure S5. Mean hydrodynamic diameter of Tf-ACAT-Se-PLGA-NPs after incubation with water, DMEM 5% FBS (Dulbecco's Modified Eagle's Medium with 5% fetal bovine serum) and plasma up to 72 h. Each value represents mean ± SD. Statistical analyses were performed by Student t-test and no significant differences were found.

Table S2. IC_{50} values (µg/mL) after 72 h of treatment

	IC_{50}				
	A375	HeLa	MCF-7	U-87	NCI/ADR-RES
Free ACAT-Se	NC	72.82*	97.38*	NC	NC
ACAT-Se-PLGA-NPs	49.56	82.57*	32.55	52.86	143.45*
Tf-ACAT-Se-PLGA-NPs	3.53	11.52	6.24	11.72	23.73

NC – not calculated, could not be determined due the low cytotoxicity.
*Estimated IC_{50} based on dose-response curves.

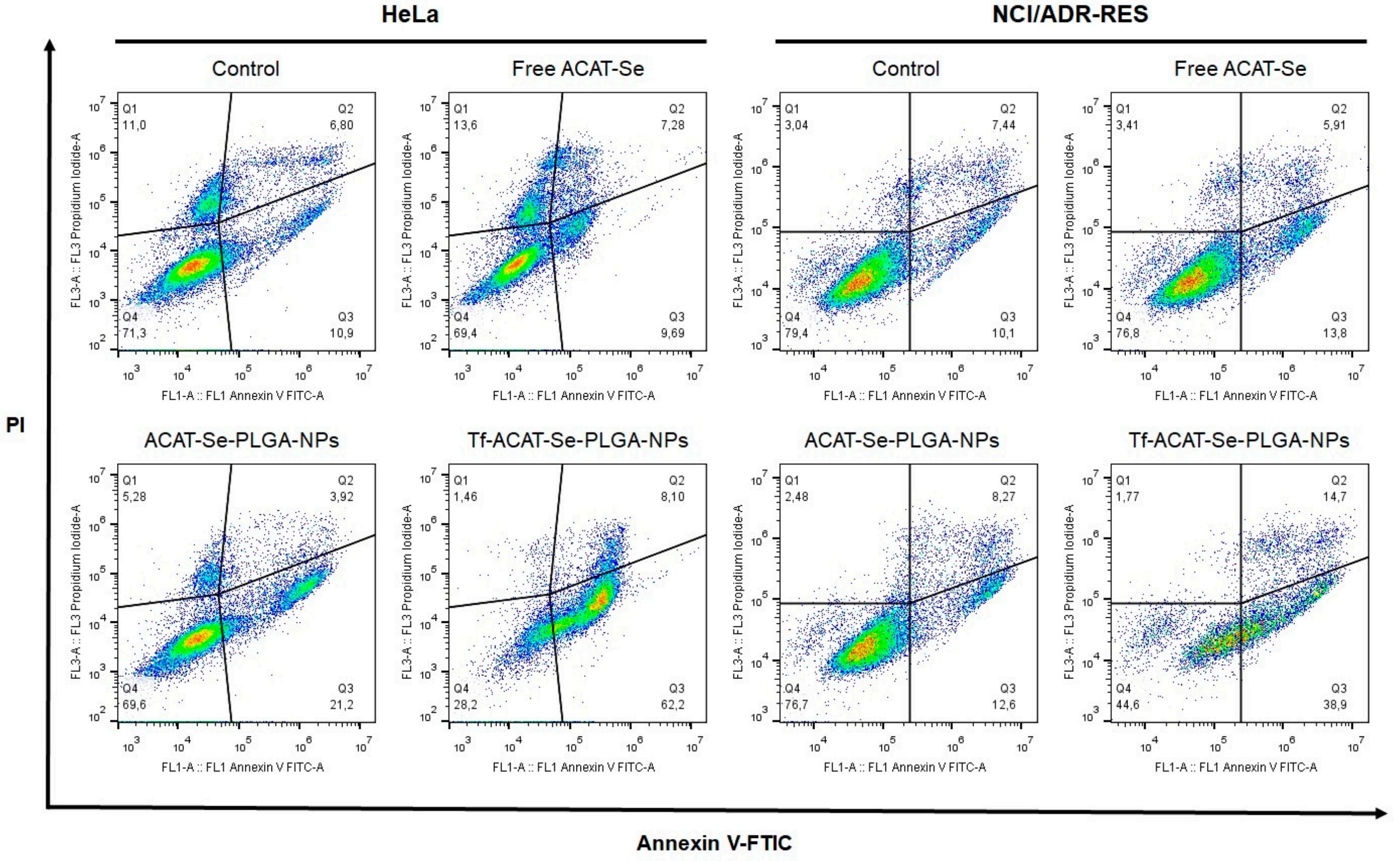

Figure S6. Scatter plots for HeLa and NCI/ADR-RES cells after 24 h incubation with free ACAT-Se, ACAT-Se-PLGA-NPs or Tf-ACAT-Se-PLGA-NPs. The results were obtained through flow cytometry after Annexin V-FITC/PI staining.

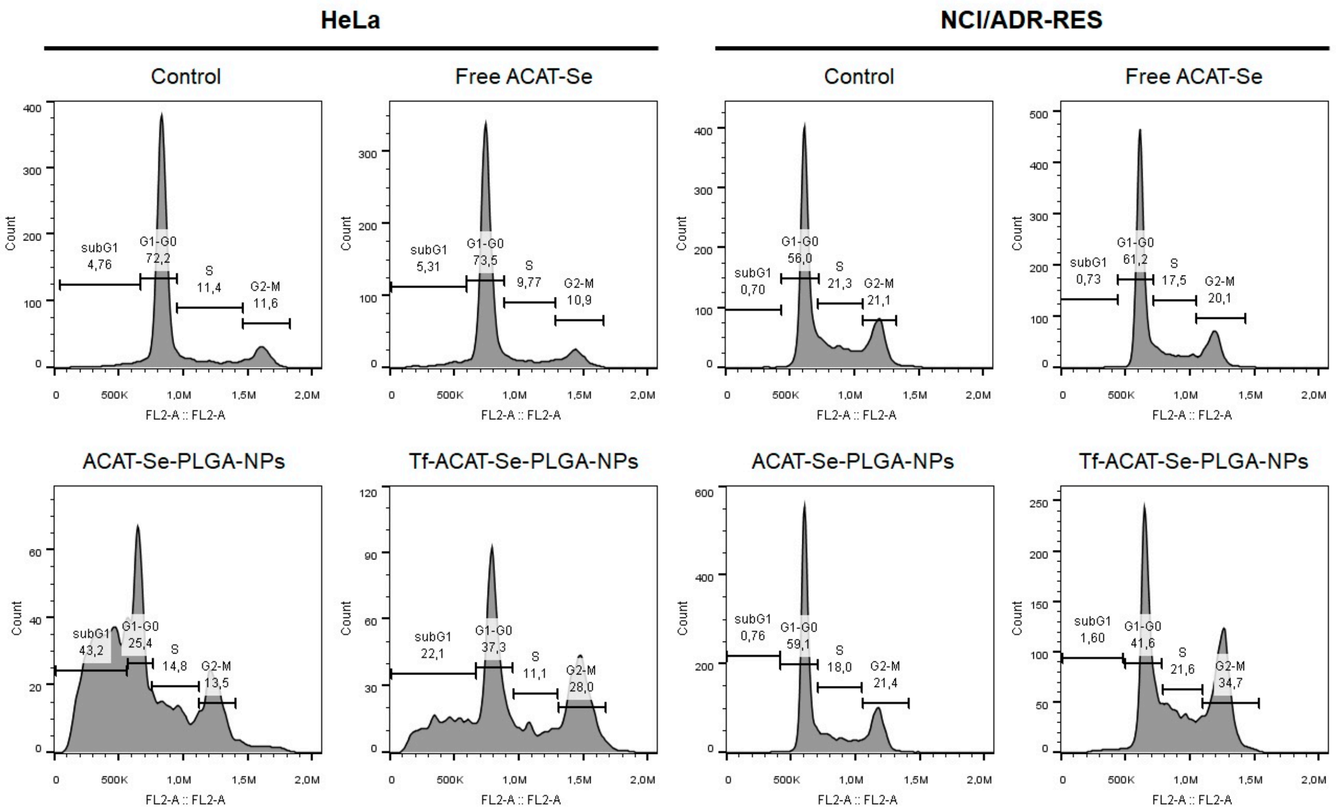

Figure S7. Histogram plots of HeLa and NCI/ADR-RES cells distribution in the different phases of the cell cycle after 24 h incubation with free ACAT-Se, ACAT-Se-PLGA-NPs or Tf-ACAT-Se-PLGA-NPs.
